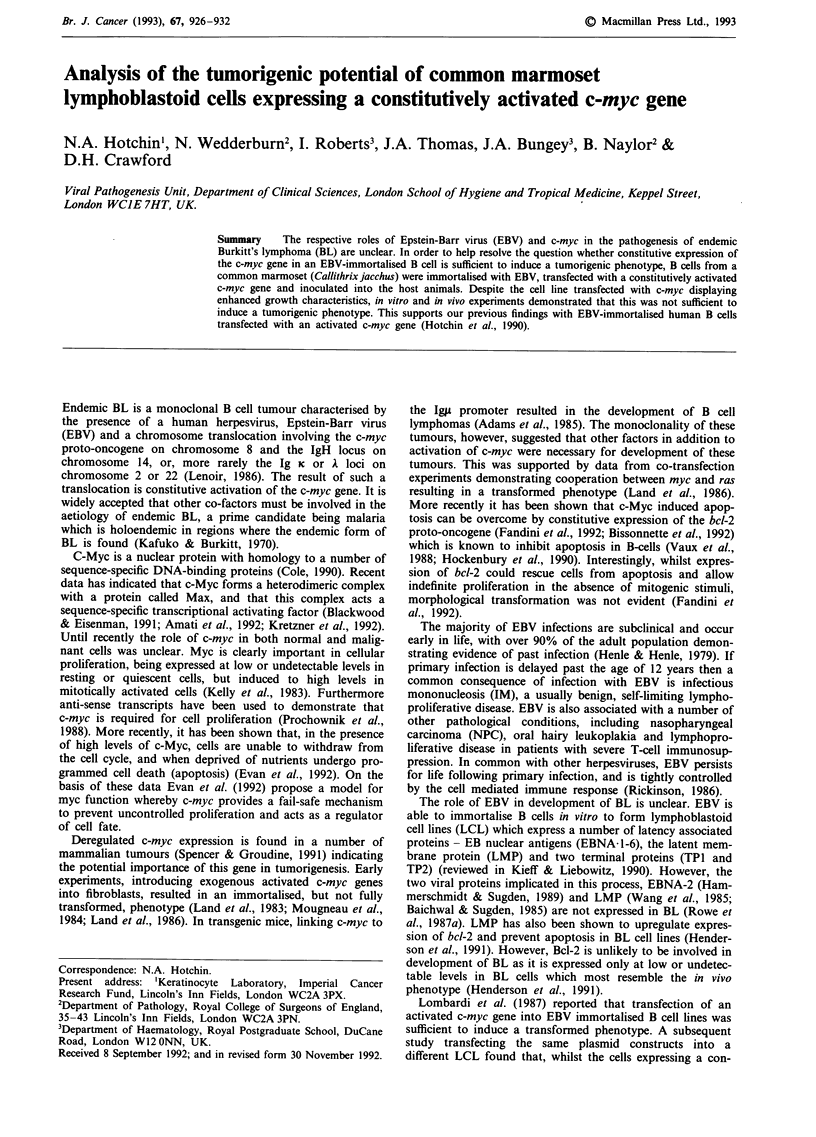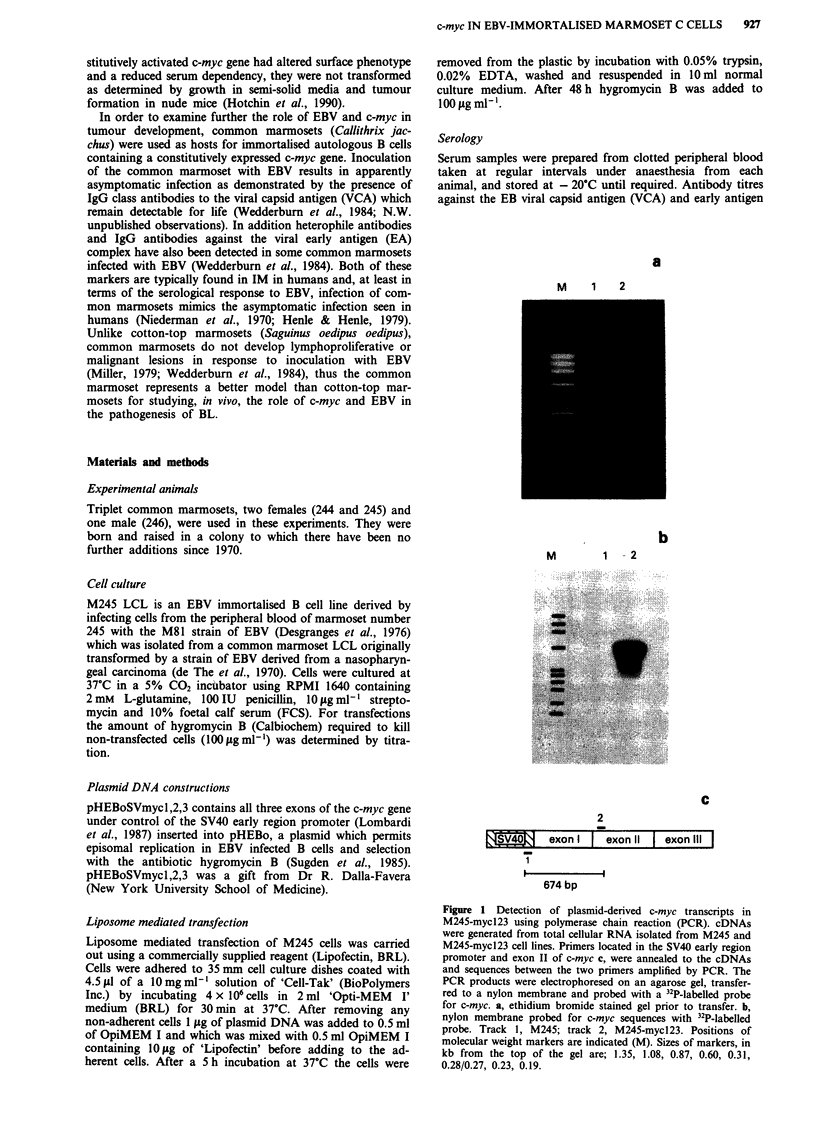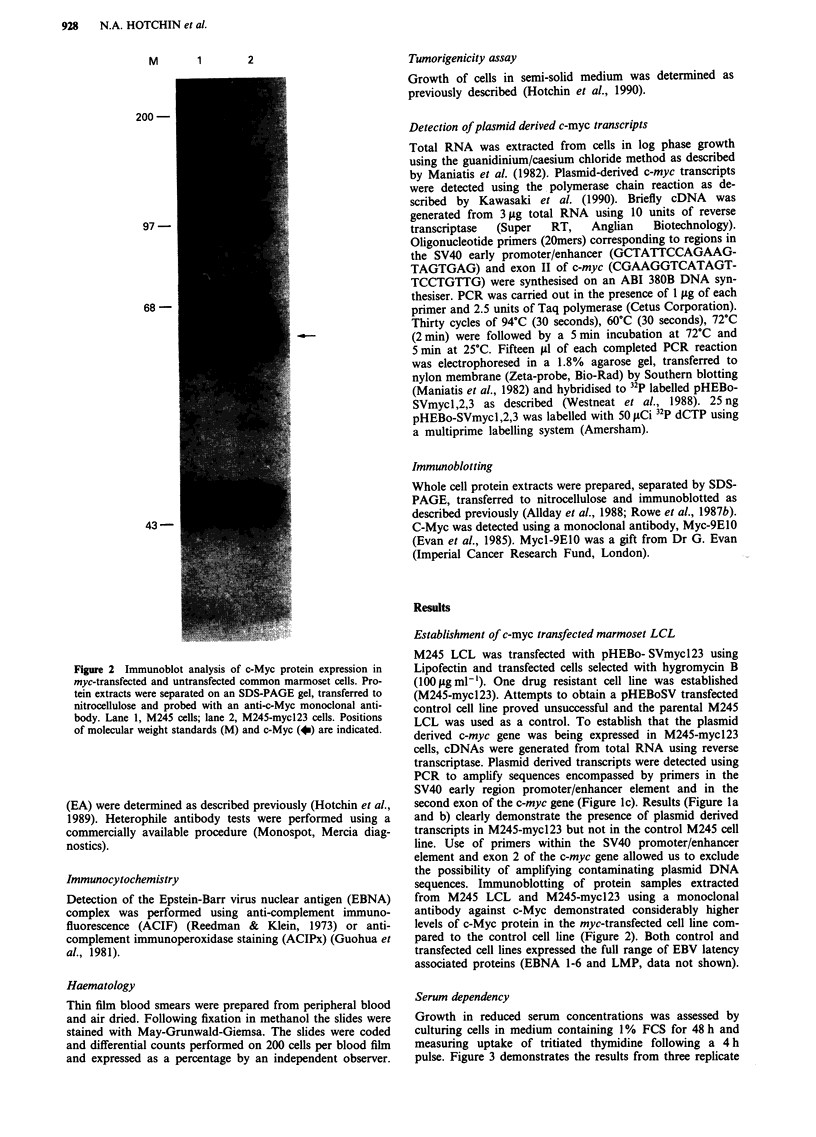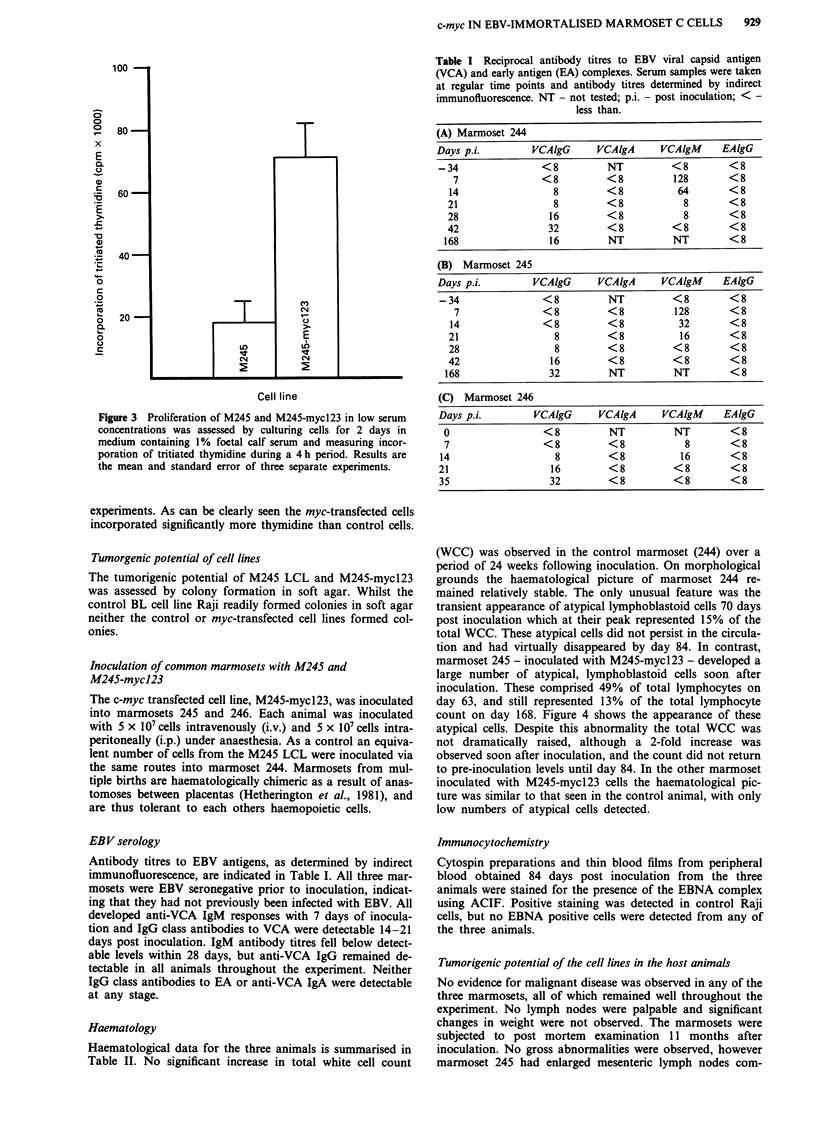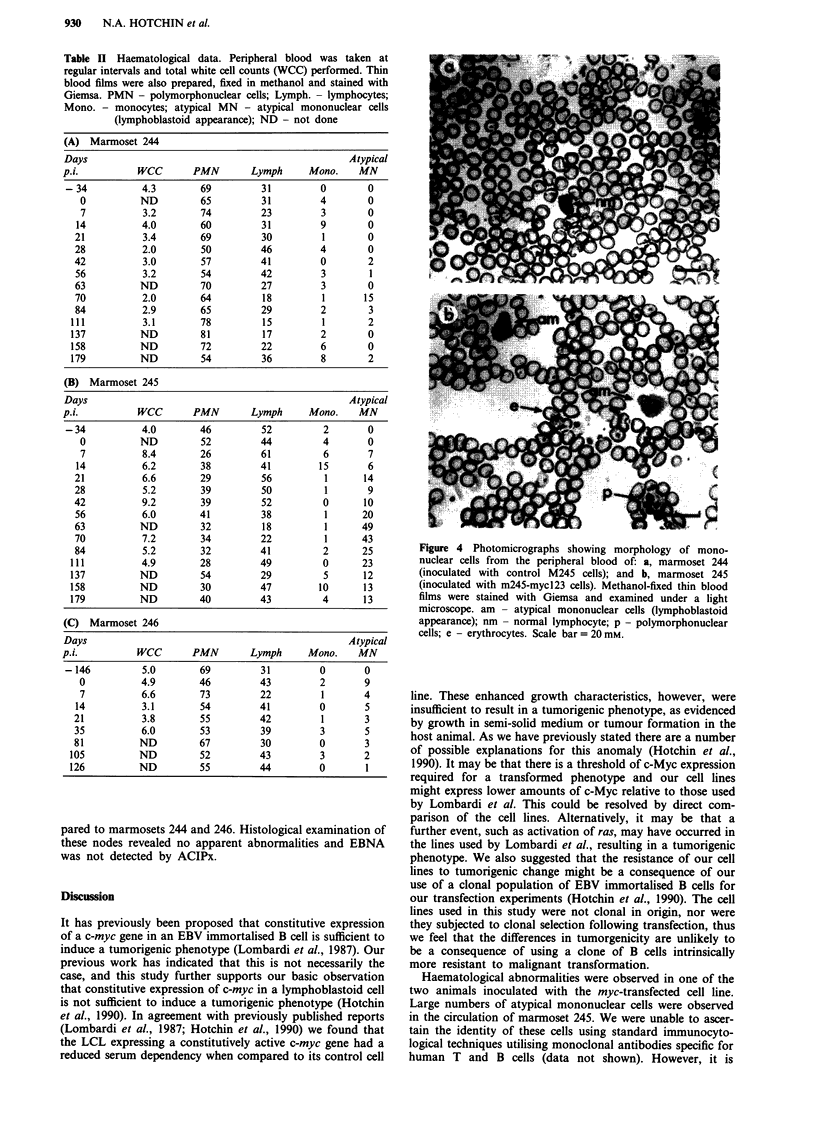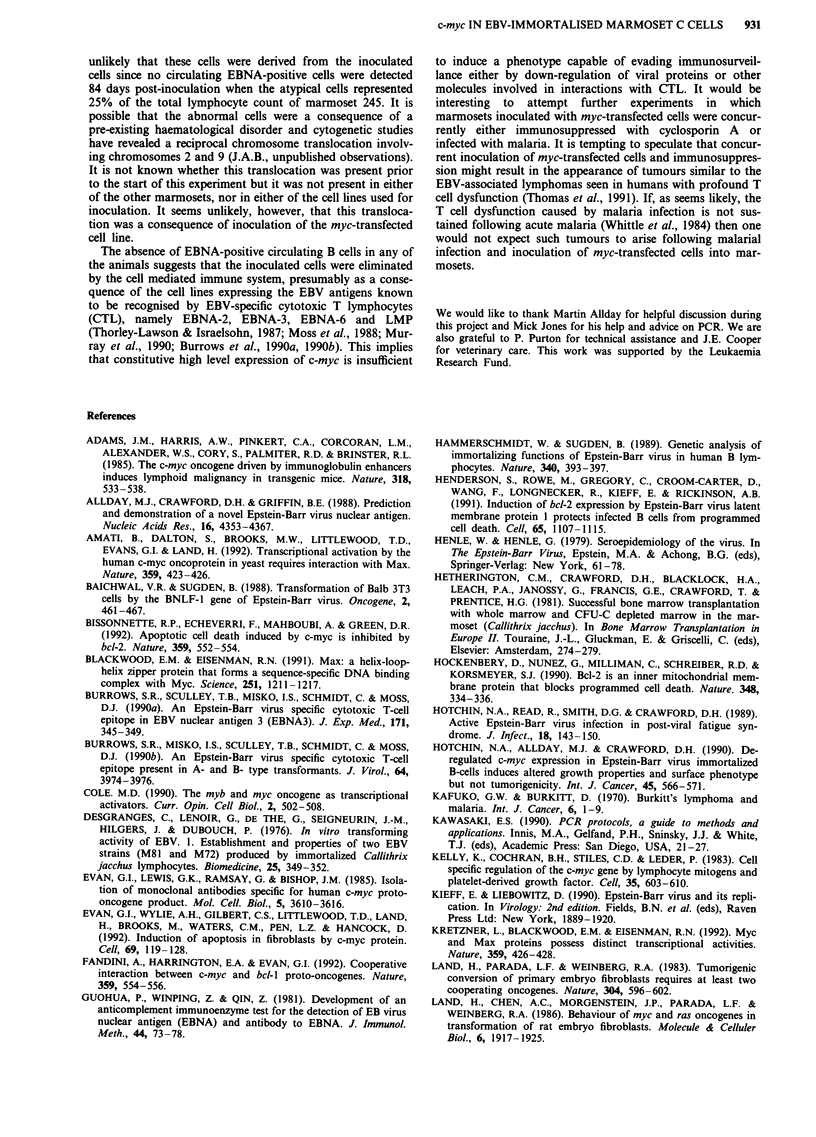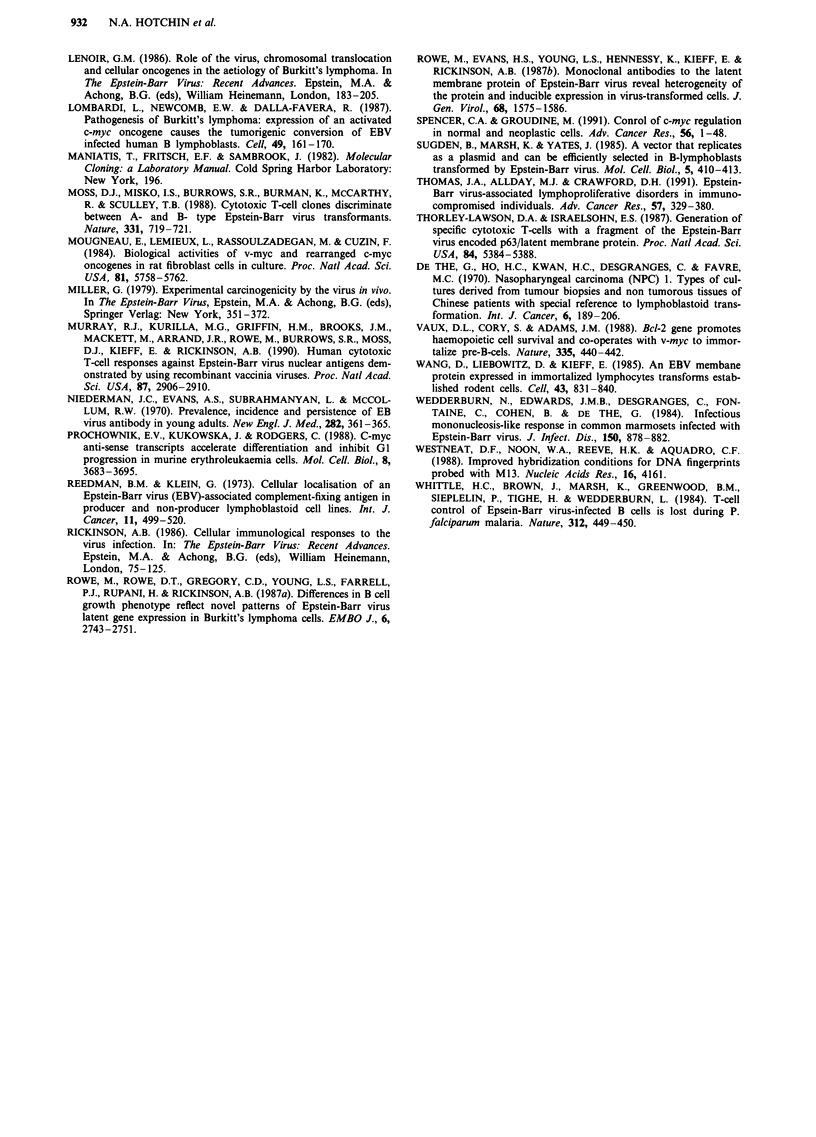# Analysis of the tumorigenic potential of common marmoset lymphoblastoid cells expressing a constitutively activated c-myc gene.

**DOI:** 10.1038/bjc.1993.172

**Published:** 1993-05

**Authors:** N. A. Hotchin, N. Wedderburn, I. Roberts, J. A. Thomas, J. A. Bungey, B. Naylor, D. H. Crawford

**Affiliations:** Department of Clinical Sciences, London School of Hygiene and Tropical Medicine, UK.

## Abstract

**Images:**


Br. J. Cancer (1993), 67, 926-932 © Macmillan Press Ltd., 1993

### Analysis of the tumorigenic potential of common marmoset lymphoblastoid cells expressing a constitutively activated c-*myc* gene

N.A. Hotchin[1], N. Wedderburn[2], I. Roberts[3], J.A. Thomas, J.A. Bungey[3], B. Naylor[2] & D.H. Crawford

*Viral Pathogenesis Unit, Department of Clinical Sciences, London School of Hygiene and Tropical Medicine, Keppel Street, London WC1E 7HT, UK.*

**Summary** The respective roles of Epstein-Barr virus (EBV) and c-*myc* in the pathogenesis of endemic Burkitt's lymphoma (BL) are unclear. In order to help resolve the question whether constitutive expression of the c-*myc* gene in an EBV-immortalised B cell is sufficient to induce a tumorigenic phenotype, B cells from a common marmoset (*Callithrix jacchus*) were immortalised with EBV, transfected with a constitutively activated c-*myc* gene and inoculated into the host animals. Despite the cell line transfected with c-*myc* displaying enhanced growth characteristics, *in vitro* and *in vivo* experiments demonstrated that this was not sufficient to induce a tumorigenic phenotype. This supports our previous findings with EBV-immortalised human B cells transfected with an activated c-*myc* gene (Hotchin *et al.*, 1990).

Endemic BL is a monoclonal B cell tumour characterised by the presence of a human herpesvirus, Epstein-Barr virus (EBV) and a chromosome translocation involving the c-*myc* proto-oncogene on chromosome 8 and the IgH locus on chromosome 14, or, more rarely the Ig κ or λ loci on chromosome 2 or 22 (Lenoir, 1986). The result of such a translocation is constitutive activation of the c-*myc* gene. It is widely accepted that other co-factors must be involved in the aetiology of endemic BL, a prime candidate being malaria which is holoendemic in regions where the endemic form of BL is found (Kafuko & Burkitt, 1970).

C-Myc is a nuclear protein with homology to a number of sequence-specific DNA-binding proteins (Cole, 1990). Recent data has indicated that c-Myc forms a heterodimeric complex with a protein called Max, and that this complex acts a sequence-specific transcriptional activating factor (Blackwood & Eisenman, 1991; Amati *et al.*, 1992; Kretzner *et al.*, 1992). Until recently the role of c-*myc* in both normal and malignant cells was unclear. Myc is clearly important in cellular proliferation, being expressed at low or undetectable levels in resting or quiescent cells, but induced to high levels in mitotically activated cells (Kelly *et al.*, 1983). Furthermore anti-sense transcripts have been used to demonstrate that c-*myc* is required for cell proliferation (Prochownik *et al.*, 1988). More recently, it has been shown that, in the presence of high levels of c-Myc, cells are unable to withdraw from the cell cycle, and when deprived of nutrients undergo programmed cell death (apoptosis) (Evan *et al.*, 1992). On the basis of these data Evan *et al.* (1992) propose a model for myc function whereby c-*myc* provides a fail-safe mechanism to prevent uncontrolled proliferation and acts as a regulator of cell fate.

Deregulated c-*myc* expression is found in a number of mammalian tumours (Spencer & Groudine, 1991) indicating the potential importance of this gene in tumorigenesis. Early experiments, introducing exogenous activated c-*myc* genes into fibroblasts, resulted in an immortalised, but not fully transformed, phenotype (Land *et al.*, 1983; Mougneau *et al.*, 1984; Land *et al.*, 1986). In transgenic mice, linking c-*myc* to the Igμ promoter resulted in the development of B cell lymphomas (Adams *et al.*, 1985). The monoclonality of these tumours, however, suggested that other factors in addition to activation of c-*myc* were necessary for development of these tumours. This was supported by data from co-transfection experiments demonstrating cooperation between *myc* and *ras* resulting in a transformed phenotype (Land *et al.*, 1986). More recently it has been shown that c-Myc induced apoptosis can be overcome by constitutive expression of the *bcl*-2 proto-oncogene (Fandini *et al.*, 1992; Bissonnette *et al.*, 1992) which is known to inhibit apoptosis in B-cells (Vaux *et al.*, 1988; Hockenbury *et al.*, 1990). Interestingly, whilst expression of *bcl*-2 could rescue cells from apoptosis and allow indefinite proliferation in the absence of mitogenic stimuli, morphological transformation was not evident (Fandini *et al.*, 1992).

The majority of EBV infections are subclinical and occur early in life, with over 90% of the adult population demonstrating evidence of past infection (Henle & Henle, 1979). If primary infection is delayed past the age of 12 years then a common consequence of infection with EBV is infectious mononucleosis (IM), a usually benign, self-limiting lymphoproliferative disease. EBV is also associated with a number of other pathological conditions, including nasopharyngeal carcinoma (NPC), oral hairy leukoplakia and lymphoproliferative disease in patients with severe T-cell immunosuppression. In common with other herpesviruses, EBV persists for life following primary infection, and is tightly controlled by the cell mediated immune response (Rickinson, 1986).

The role of EBV in development of BL is unclear. EBV is able to immortalise B cells *in vitro* to form lymphoblastoid cell lines (LCL) which express a number of latency associated proteins – EB nuclear antigens (EBNA·1-6), the latent membrane protein (LMP) and two terminal proteins (TP1 and TP2) (reviewed in Kieff & Liebowitz, 1990). However, the two viral proteins implicated in this process, EBNA-2 (Hammerschmidt & Sugden, 1989) and LMP (Wang *et al.*, 1985; Baichwal & Sugden, 1985) are not expressed in BL (Rowe *et al.*, 1987*a*). LMP has also been shown to upregulate expression of *bcl*-2 and prevent apoptosis in BL cell lines (Henderson *et al.*, 1991). However, Bcl-2 is unlikely to be involved in development of BL as it is expressed only at low or undetectable levels in BL cells which most resemble the *in vivo* phenotype (Henderson *et al.*, 1991).

Lombardi *et al.* (1987) reported that transfection of an activated c-*myc* gene into EBV immortalised B cell lines was sufficient to induce a transformed phenotype. A subsequent study transfecting the same plasmid constructs into a different LCL found that, whilst the cells expressing a con-

Correspondence: N.A. Hotchin.
Present address: [1]Keratinocyte Laboratory, Imperial Cancer Research Fund, Lincoln's Inn Fields, London WC2A 3PX.
[2]Department of Pathology, Royal College of Surgeons of England, 35-43 Lincoln's Inn Fields, London WC2A 3PN.
[3]Department of Haematology, Royal Postgraduate School, DuCane Road, London W12 0NN, UK.
Received 8 September 1992; and in revised form 30 November 1992.

stitutively activated c-*myc* gene had altered surface phenotype and a reduced serum dependency, they were not transformed as determined by growth in semi-solid media and tumour formation in nude mice (Hotchin *et al.*, 1990).

In order to examine further the role of EBV and c-*myc* in tumour development, common marmosets (*Callithrix jacchus*) were used as hosts for immortalised autologous B cells containing a constitutively expressed c-*myc* gene. Inoculation of the common marmoset with EBV results in apparently asymptomatic infection as demonstrated by the presence of IgG class antibodies to the viral capsid antigen (VCA) which remain detectable for life (Wedderburn *et al.*, 1984; N.W. unpublished observations). In addition heterophile antibodies and IgG antibodies against the viral early antigen (EA) complex have also been detected in some common marmosets infected with EBV (Wedderburn *et al.*, 1984). Both of these markers are typically found in IM in humans and, at least in terms of the serological response to EBV, infection of common marmosets mimics the asymptomatic infection seen in humans (Niederman *et al.*, 1970; Henle & Henle, 1979). Unlike cotton-top marmosets (*Saguinus oedipus oedipus*), common marmosets do not develop lymphoproliferative or malignant lesions in response to inoculation with EBV (Miller, 1979; Wedderburn *et al.*, 1984), thus the common marmoset represents a better model than cotton-top marmosets for studying, *in vivo*, the role of c-*myc* and EBV in the pathogenesis of BL.

### Materials and methods

### *Experimental animals*

Triplet common marmosets, two females (244 and 245) and one male (246), were used in these experiments. They were born and raised in a colony to which there have been no further additions since 1970.

### *Cell culture*

M245 LCL is an EBV immortalised B cell line derived by infecting cells from the peripheral blood of marmoset number 245 with the M81 strain of EBV (Desgranges *et al.*, 1976) which was isolated from a common marmoset LCL originally transformed by a strain of EBV derived from a nasopharyngeal carcinoma (de The *et al.*, 1970). Cells were cultured at 37°C in a 5% $CO_2$ incubator using RPMI 1640 containing 2 mM L-glutamine, 100 IU penicillin, 10 $\mu g\ ml^{-1}$ streptomycin and 10% foetal calf serum (FCS). For transfections the amount of hygromycin B (Calbiochem) required to kill non-transfected cells (100 $\mu g\ ml^{-1}$) was determined by titration.

### *Plasmid DNA constructions*

pHEBoSVmyc1,2,3 contains all three exons of the c-*myc* gene under control of the SV40 early region promoter (Lombardi *et al.*, 1987) inserted into pHEBo, a plasmid which permits episomal replication in EBV infected B cells and selection with the antibiotic hygromycin B (Sugden *et al.*, 1985). pHEBoSVmyc1,2,3 was a gift from Dr R. Dalla-Favera (New York University School of Medicine).

### *Liposome mediated transfection*

Liposome mediated transfection of M245 cells was carried out using a commercially supplied reagent (Lipofectin, BRL). Cells were adhered to 35 mm cell culture dishes coated with 4.5 $\mu l$ of a 10 $mg\ ml^{-1}$ solution of 'Cell-Tak' (BioPolymers Inc.) by incubating $4 \times 10^6$ cells in 2 ml 'Opti-MEM I' medium (BRL) for 30 min at 37°C. After removing any non-adherent cells 1 $\mu g$ of plasmid DNA was added to 0.5 ml of OpiMEM I and which was mixed with 0.5 ml OpiMEM I containing 10 $\mu g$ of 'Lipofectin' before adding to the adherent cells. After a 5 h incubation at 37°C the cells were removed from the plastic by incubation with 0.05% trypsin, 0.02% EDTA, washed and resuspended in 10 ml normal culture medium. After 48 h hygromycin B was added to 100 $\mu g\ ml^{-1}$.

### *Serology*

Serum samples were prepared from clotted peripheral blood taken at regular intervals under anaesthesia from each animal, and stored at − 20°C until required. Antibody titres against the EB viral capsid antigen (VCA) and early antigen

**Figure 1** Detection of plasmid-derived c-*myc* transcripts in M245-myc123 using polymerase chain reaction (PCR). cDNAs were generated from total cellular RNA isolated from M245 and M245-myc123 cell lines. Primers located in the SV40 early region promoter and exon II of c-*myc* **c**, were annealed to the cDNAs and sequences between the two primers amplified by PCR. The PCR products were electrophoresed on an agarose gel, transferred to a nylon membrane and probed with a $^{32}P$-labelled probe for c-*myc*. **a**, ethidium bromide stained gel prior to transfer. **b**, nylon membrane probed for c-*myc* sequences with $^{32}P$-labelled probe. Track 1, M245; track 2, M245-myc123. Positions of molecular weight markers are indicated (M). Sizes of markers, in kb from the top of the gel are; 1.35, 1.08, 0.87, 0.60, 0.31, 0.28/0.27, 0.23, 0.19.

**Figure 2** Immunoblot analysis of c-Myc protein expression in *myc*-transfected and untransfected common marmoset cells. Protein extracts were separated on an SDS-PAGE gel, transferred to nitrocellulose and probed with an anti-c-Myc monoclonal antibody. Lane 1, M245 cells; lane 2, M245-myc123 cells. Positions of molecular weight standards (M) and c-Myc (←) are indicated.

(EA) were determined as described previously (Hotchin *et al.*, 1989). Heterophile antibody tests were performed using a commercially available procedure (Monospot, Mercia diagnostics).

*Immunocytochemistry*

Detection of the Epstein-Barr virus nuclear antigen (EBNA) complex was performed using anti-complement immunofluorescence (ACIF) (Reedman & Klein, 1973) or anti-complement immunoperoxidase staining (ACIPx) (Guohua *et al.*, 1981).

*Haematology*

Thin film blood smears were prepared from peripheral blood and air dried. Following fixation in methanol the slides were stained with May-Grunwald-Giemsa. The slides were coded and differential counts performed on 200 cells per blood film and expressed as a percentage by an independent observer.

*Tumorigenicity assay*

Growth of cells in semi-solid medium was determined as previously described (Hotchin *et al.*, 1990).

*Detection of plasmid derived* c-myc *transcripts*

Total RNA was extracted from cells in log phase growth using the guanidinium/caesium chloride method as described by Maniatis *et al.* (1982). Plasmid-derived c-*myc* transcripts were detected using the polymerase chain reaction as described by Kawasaki *et al.* (1990). Briefly cDNA was generated from 3 μg total RNA using 10 units of reverse transcriptase (Super RT, Anglian Biotechnology). Oligonucleotide primers (20mers) corresponding to regions in the SV40 early promoter/enhancer (GCTATTCCAGAAGTAGTGAG) and exon II of c-*myc* (CGAAGGTCATAGTTCCTGTTG) were synthesised on an ABI 380B DNA synthesiser. PCR was carried out in the presence of 1 μg of each primer and 2.5 units of Taq polymerase (Cetus Corporation). Thirty cycles of 94°C (30 seconds), 60°C (30 seconds), 72°C (2 min) were followed by a 5 min incubation at 72°C and 5 min at 25°C. Fifteen μl of each completed PCR reaction was electrophoresed in a 1.8% agarose gel, transferred to nylon membrane (Zeta-probe, Bio-Rad) by Southern blotting (Maniatis *et al.*, 1982) and hybridised to $^{32}P$ labelled pHEBo-SVmyc1,2,3 as described (Westneat *et al.*, 1988). 25 ng pHEBo-SVmyc1,2,3 was labelled with 50 μCi $^{32}P$ dCTP using a multiprime labelling system (Amersham).

*Immunoblotting*

Whole cell protein extracts were prepared, separated by SDS-PAGE, transferred to nitrocellulose and immunoblotted as described previously (Allday *et al.*, 1988; Rowe *et al.*, 1987*b*). C-Myc was detected using a monoclonal antibody, Myc-9E10 (Evan *et al.*, 1985). Myc1-9E10 was a gift from Dr G. Evan (Imperial Cancer Research Fund, London).

### Results

*Establishment of c-*myc *transfected marmoset LCL*

M245 LCL was transfected with pHEBo-SVmyc123 using Lipofectin and transfected cells selected with hygromycin B (100 μg ml$^{-1}$). One drug resistant cell line was established (M245-myc123). Attempts to obtain a pHEBoSV transfected control cell line proved unsuccessful and the parental M245 LCL was used as a control. To establish that the plasmid derived c-*myc* gene was being expressed in M245-myc123 cells, cDNAs were generated from total RNA using reverse transcriptase. Plasmid derived transcripts were detected using PCR to amplify sequences encompassed by primers in the SV40 early region promoter/enhancer element and in the second exon of the c-*myc* gene (Figure 1c). Results (Figure 1a and b) clearly demonstrate the presence of plasmid derived transcripts in M245-myc123 but not in the control M245 cell line. Use of primers within the SV40 promoter/enhancer element and exon 2 of the c-*myc* gene allowed us to exclude the possibility of amplifying contaminating plasmid DNA sequences. Immunoblotting of protein samples extracted from M245 LCL and M245-myc123 using a monoclonal antibody against c-Myc demonstrated considerably higher levels of c-Myc protein in the *myc*-transfected cell line compared to the control cell line (Figure 2). Both control and transfected cell lines expressed the full range of EBV latency associated proteins (EBNA 1-6 and LMP, data not shown).

*Serum dependency*

Growth in reduced serum concentrations was assessed by culturing cells in medium containing 1% FCS for 48 h and measuring uptake of tritiated thymidine following a 4 h pulse. Figure 3 demonstrates the results from three replicate

**Figure 3** Proliferation of M245 and M245-myc123 in low serum concentrations was assessed by culturing cells for 2 days in medium containing 1% foetal calf serum and measuring incorporation of tritiated thymidine during a 4 h period. Results are the mean and standard error of three separate experiments.

**Table I** Reciprocal antibody titres to EBV viral capsid antigen (VCA) and early antigen (EA) complexes. Serum samples were taken at regular time points and antibody titres determined by indirect immunofluorescence. NT – not tested; p.i. – post inoculation; < – less than.

| (A) Marmoset 244 | | | | |
|---|---|---|---|---|
| *Days p.i.* | *VCAIgG* | *VCAIgA* | *VCAIgM* | *EAIgG* |
| −34 | <8 | NT | <8 | <8 |
| 7 | <8 | <8 | 128 | <8 |
| 14 | 8 | <8 | 64 | <8 |
| 21 | 8 | <8 | 8 | <8 |
| 28 | 16 | <8 | 8 | <8 |
| 42 | 32 | <8 | <8 | <8 |
| 168 | 16 | NT | NT | <8 |
| **(B)** Marmoset 245 | | | | |
| *Days p.i.* | *VCAIgG* | *VCAIgA* | *VCAIgM* | *EAIgG* |
| −34 | <8 | NT | <8 | <8 |
| 7 | <8 | <8 | 128 | <8 |
| 14 | <8 | <8 | 32 | <8 |
| 21 | 8 | <8 | 16 | <8 |
| 28 | 8 | <8 | <8 | <8 |
| 42 | 16 | <8 | <8 | <8 |
| 168 | 32 | NT | NT | <8 |
| (C) Marmoset 246 | | | | |
| *Days p.i.* | *VCAIgG* | *VCAIgA* | *VCAIgM* | *EAIgG* |
| 0 | <8 | NT | NT | <8 |
| 7 | <8 | <8 | 8 | <8 |
| 14 | 8 | <8 | 16 | <8 |
| 21 | 16 | <8 | <8 | <8 |
| 35 | 32 | <8 | <8 | <8 |

experiments. As can be clearly seen the *myc*-transfected cells incorporated significantly more thymidine than control cells.

### *Tumorgenic potential of cell lines*

The tumorigenic potential of M245 LCL and M245-myc123 was assessed by colony formation in soft agar. Whilst the control BL cell line Raji readily formed colonies in soft agar neither the control or *myc*-transfected cell lines formed colonies.

### *Inoculation of common marmosets with M245 and M245-myc123*

The c-*myc* transfected cell line, M245-myc123, was inoculated into marmosets 245 and 246. Each animal was inoculated with $5 \times 10^7$ cells intravenously (i.v.) and $5 \times 10^7$ cells intraperitoneally (i.p.) under anaesthesia. As a control an equivalent number of cells from the M245 LCL were inoculated via the same routes into marmoset 244. Marmosets from multiple births are haematologically chimeric as a result of anastomoses between placentas (Hetherington *et al.*, 1981), and are thus tolerant to each others haemopoietic cells.

### *EBV serology*

Antibody titres to EBV antigens, as determined by indirect immunofluorescence, are indicated in Table I. All three marmosets were EBV seronegative prior to inoculation, indicating that they had not previously been infected with EBV. All developed anti-VCA IgM responses with 7 days of inoculation and IgG class antibodies to VCA were detectable 14–21 days post inoculation. IgM antibody titres fell below detectable levels within 28 days, but anti-VCA IgG remained detectable in all animals throughout the experiment. Neither IgG class antibodies to EA or anti-VCA IgA were detectable at any stage.

### *Haematology*

Haematological data for the three animals is summarised in Table II. No significant increase in total white cell count (WCC) was observed in the control marmoset (244) over a period of 24 weeks following inoculation. On morphological grounds the haematological picture of marmoset 244 remained relatively stable. The only unusual feature was the transient appearance of atypical lymphoblastoid cells 70 days post inoculation which at their peak represented 15% of the total WCC. These atypical cells did not persist in the circulation and had virtually disappeared by day 84. In contrast, marmoset 245 – inoculated with M245-myc123 – developed a large number of atypical, lymphoblastoid cells soon after inoculation. These comprised 49% of total lymphocytes on day 63, and still represented 13% of the total lymphocyte count on day 168. Figure 4 shows the appearance of these atypical cells. Despite this abnormality the total WCC was not dramatically raised, although a 2-fold increase was observed soon after inoculation, and the count did not return to pre-inoculation levels until day 84. In the other marmoset inoculated with M245-myc123 cells the haematological picture was similar to that seen in the control animal, with only low numbers of atypical cells detected.

### *Immunocytochemistry*

Cytospin preparations and thin blood films from peripheral blood obtained 84 days post inoculation from the three animals were stained for the presence of the EBNA complex using ACIF. Positive staining was detected in control Raji cells, but no EBNA positive cells were detected from any of the three animals.

### *Tumorigenic potential of the cell lines in the host animals*

No evidence for malignant disease was observed in any of the three marmosets, all of which remained well throughout the experiment. No lymph nodes were palpable and significant changes in weight were not observed. The marmosets were subjected to post mortem examination 11 months after inoculation. No gross abnormalities were observed, however marmoset 245 had enlarged mesenteric lymph nodes com-

**Table II** Haematological data. Peripheral blood was taken at regular intervals and total white cell counts (WCC) performed. Thin blood films were also prepared, fixed in methanol and stained with Giemsa. PMN – polymorphonuclear cells; Lymph. – lymphocytes; Mono. – monocytes; atypical MN – atypical mononuclear cells (lymphoblastoid appearance); ND – not done

(A) Marmoset 244

| *Days p.i.* | *WCC* | *PMN* | *Lymph* | *Mono.* | *Atypical MN* |
|---|---|---|---|---|---|
| −34 | 4.3 | 69 | 31 | 0 | 0 |
| 0 | ND | 65 | 31 | 4 | 0 |
| 7 | 3.2 | 74 | 23 | 3 | 0 |
| 14 | 4.0 | 60 | 31 | 9 | 0 |
| 21 | 3.4 | 69 | 30 | 1 | 0 |
| 28 | 2.0 | 50 | 46 | 4 | 0 |
| 42 | 3.0 | 57 | 41 | 0 | 2 |
| 56 | 3.2 | 54 | 42 | 3 | 1 |
| 63 | ND | 70 | 27 | 3 | 0 |
| 70 | 2.0 | 64 | 18 | 1 | 15 |
| 84 | 2.9 | 65 | 29 | 2 | 3 |
| 111 | 3.1 | 78 | 15 | 1 | 2 |
| 137 | ND | 81 | 17 | 2 | 0 |
| 158 | ND | 72 | 22 | 6 | 0 |
| 179 | ND | 54 | 36 | 8 | 2 |

(B) Marmoset 245

| *Days p.i.* | *WCC* | *PMN* | *Lymph* | *Mono.* | *Atypical MN* |
|---|---|---|---|---|---|
| −34 | 4.0 | 46 | 52 | 2 | 0 |
| 0 | ND | 52 | 44 | 4 | 0 |
| 7 | 8.4 | 26 | 61 | 6 | 7 |
| 14 | 6.2 | 38 | 41 | 15 | 6 |
| 21 | 6.6 | 29 | 56 | 1 | 14 |
| 28 | 5.2 | 39 | 50 | 1 | 9 |
| 42 | 9.2 | 39 | 52 | 0 | 10 |
| 56 | 6.0 | 41 | 38 | 1 | 20 |
| 63 | ND | 32 | 18 | 1 | 49 |
| 70 | 7.2 | 34 | 22 | 1 | 43 |
| 84 | 5.2 | 32 | 41 | 2 | 25 |
| 111 | 4.9 | 28 | 49 | 0 | 23 |
| 137 | ND | 54 | 29 | 5 | 12 |
| 158 | ND | 30 | 47 | 10 | 13 |
| 179 | ND | 40 | 43 | 4 | 13 |

(C) Marmoset 246

| *Days p.i.* | *WCC* | *PMN* | *Lymph* | *Mono.* | *Atypical MN* |
|---|---|---|---|---|---|
| −146 | 5.0 | 69 | 31 | 0 | 0 |
| 0 | 4.9 | 46 | 43 | 2 | 9 |
| 7 | 6.6 | 73 | 22 | 1 | 4 |
| 14 | 3.1 | 54 | 41 | 0 | 5 |
| 21 | 3.8 | 55 | 42 | 1 | 3 |
| 35 | 6.0 | 53 | 39 | 3 | 5 |
| 81 | ND | 67 | 30 | 0 | 3 |
| 105 | ND | 52 | 43 | 3 | 2 |
| 126 | ND | 55 | 44 | 0 | 1 |

**Figure 4** Photomicrographs showing morphology of mononuclear cells from the peripheral blood of: **a**, marmoset 244 (inoculated with control M245 cells); and **b**, marmoset 245 (inoculated with m245-myc123 cells). Methanol-fixed thin blood films were stained with Giemsa and examined under a light microscope. am – atypical mononuclear cells (lymphoblastoid appearance); nm – normal lymphocyte; p – polymorphonuclear cells; e – erythrocytes. Scale bar = 20 mM.

pared to marmosets 244 and 246. Histological examination of these nodes revealed no apparent abnormalities and EBNA was not detected by ACIPx.

### Discussion

It has previously been proposed that constitutive expression of a c-*myc* gene in an EBV immortalised B cell is sufficient to induce a tumorigenic phenotype (Lombardi *et al.*, 1987). Our previous work has indicated that this is not necessarily the case, and this study further supports our basic observation that constitutive expression of c-*myc* in a lymphoblastoid cell is not sufficient to induce a tumorigenic phenotype (Hotchin *et al.*, 1990). In agreement with previously published reports (Lombardi *et al.*, 1987; Hotchin *et al.*, 1990) we found that the LCL expressing a constitutively active c-*myc* gene had a reduced serum dependency when compared to its control cell line. These enhanced growth characteristics, however, were insufficient to result in a tumorigenic phenotype, as evidenced by growth in semi-solid medium or tumour formation in the host animal. As we have previously stated there are a number of possible explanations for this anomaly (Hotchin *et al.*, 1990). It may be that there is a threshold of c-Myc expression required for a transformed phenotype and our cell lines might express lower amounts of c-Myc relative to those used by Lombardi *et al.* This could be resolved by direct comparison of the cell lines. Alternatively, it may be that a further event, such as activation of *ras*, may have occurred in the lines used by Lombardi *et al.*, resulting in a tumorigenic phenotype. We also suggested that the resistance of our cell lines to tumorigenic change might be a consequence of our use of a clonal population of EBV immortalised B cells for our transfection experiments (Hotchin *et al.*, 1990). The cell lines used in this study were not clonal in origin, nor were they subjected to clonal selection following transfection, thus we feel that the differences in tumorgenicity are unlikely to be a consequence of using a clone of B cells intrinsically more resistant to malignant transformation.

Haematological abnormalities were observed in one of the two animals inoculated with the *myc*-transfected cell line. Large numbers of atypical mononuclear cells were observed in the circulation of marmoset 245. We were unable to ascertain the identity of these cells using standard immunocytological techniques utilising monoclonal antibodies specific for human T and B cells (data not shown). However, it is unlikely that these cells were derived from the inoculated cells since no circulating EBNA-positive cells were detected 84 days post-inoculation when the atypical cells represented 25% of the total lymphocyte count of marmoset 245. It is possible that the abnormal cells were a consequence of a pre-existing haematological disorder and cytogenetic studies have revealed a reciprocal chromosome translocation involving chromosomes 2 and 9 (J.A.B., unpublished observations). It is not known whether this translocation was present prior to the start of this experiment but it was not present in either of the other marmosets, nor in either of the cell lines used for inoculation. It seems unlikely, however, that this translocation was a consequence of inoculation of the *myc*-transfected cell line.

The absence of EBNA-positive circulating B cells in any of the animals suggests that the inoculated cells were eliminated by the cell mediated immune system, presumably as a consequence of the cell lines expressing the EBV antigens known to be recognised by EBV-specific cytotoxic T lymphocytes (CTL), namely EBNA-2, EBNA-3, EBNA-6 and LMP (Thorley-Lawson & Israelsohn, 1987; Moss *et al*., 1988; Murray *et al*., 1990; Burrows *et al*., 1990*a*, 1990*b*). This implies that constitutive high level expression of c-*myc* is insufficient to induce a phenotype capable of evading immunosurveillance either by down-regulation of viral proteins or other molecules involved in interactions with CTL. It would be interesting to attempt further experiments in which marmosets inoculated with *myc*-transfected cells were concurrently either immunosuppressed with cyclosporin A or infected with malaria. It is tempting to speculate that concurrent inoculation of *myc*-transfected cells and immunosuppression might result in the appearance of tumours similar to the EBV-associated lymphomas seen in humans with profound T cell dysfunction (Thomas *et al*., 1991). If, as seems likely, the T cell dysfunction caused by malaria infection is not sustained following acute malaria (Whittle *et al*., 1984) then one would not expect such tumours to arise following malarial infection and inoculation of *myc*-transfected cells into marmosets.

We would like to thank Martin Allday for helpful discussion during this project and Mick Jones for his help and advice on PCR. We are also grateful to P. Purton for technical assistance and J.E. Cooper for veterinary care. This work was supported by the Leukaemia Research Fund.

### References

ADAMS, J.M., HARRIS, A.W., PINKERT, C.A., CORCORAN, L.M., ALEXANDER, W.S., CORY, S., PALMITER, R.D. & BRINSTER, R.L. (1985). The c-*myc* oncogene driven by immunoglobulin enhancers induces lymphoid malignancy in transgenic mice. *Nature*, **318**, 533–538.

ALLDAY, M.J., CRAWFORD, D.H. & GRIFFIN, B.E. (1988). Prediction and demonstration of a novel Epstein-Barr virus nuclear antigen. *Nucleic Acids Res*., **16**, 4353–4367.

AMATI, B., DALTON, S., BROOKS, M.W., LITTLEWOOD, T.D., EVANS, G.I. & LAND, H. (1992). Transcriptional activation by the human c-myc oncoprotein in yeast requires interaction with Max. *Nature*, **359**, 423–426.

BAICHWAL, V.R. & SUGDEN, B. (1988). Transformation of Balb 3T3 cells by the BNLF-1 gene of Epstein-Barr virus. *Oncogene*, **2**, 461–467.

BISSONNETTE, R.P., ECHEVERRI, F., MAHBOUBI, A. & GREEN, D.R. (1992). Apoptotic cell death induced by c-myc is inhibited by *bcl*-2. *Nature*, **359**, 552–554.

BLACKWOOD, E.M. & EISENMAN, R.N. (1991). Max: a helix-loop-helix zipper protein that forms a sequence-specific DNA binding complex with Myc. *Science*, **251**, 1211–1217.

BURROWS, S.R., SCULLEY, T.B., MISKO, I.S., SCHMIDT, C. & MOSS, D.J. (1990*a*). An Epstein-Barr virus specific cytotoxic T-cell epitope in EBV nuclear antigen 3 (EBNA3). *J. Exp. Med*., **171**, 345–349.

BURROWS, S.R., MISKO, I.S., SCULLEY, T.B., SCHMIDT, C. & MOSS, D.J. (1990*b*). An Epstein-Barr virus specific cytotoxic T-cell epitope present in A- and B- type transformants. *J. Virol*., **64**, 3974–3976.

COLE. M.D. (1990). The *myb* and *myc* oncogene as transcriptional activators. *Curr. Opin. Cell Biol*., **2**, 502–508.

DESGRANGES, C., LENOIR, G., DE THE, G., SEIGNEURIN, J.-M., HILGERS, J. & DUBOUCH, P. (1976). *In vitro* transforming activity of EBV. 1. Establishment and properties of two EBV strains (M81 and M72) produced by immortalized *Callithrix jacchus* lymphocytes. *Biomedicine*, **25**, 349–352.

EVAN, G.I., LEWIS, G.K., RAMSAY, G. & BISHOP, J.M. (1985). Isolation of monoclonal antibodies specific for human c-*myc* proto-oncogene product. *Mol. Cell. Biol*., **5**, 3610–3616.

EVAN, G.I., WYLIE, A.H., GILBERT, C.S., LITTLEWOOD, T.D., LAND, H., BROOKS, M., WATERS, C.M., PEN, L.Z. & HANCOCK, D. (1992). Induction of apoptosis in fibroblasts by c-myc protein. *Cell*, **69**, 119–128.

FANDINI, A., HARRINGTON, E.A. & EVAN, G.I. (1992). Cooperative interaction between c-*myc* and *bcl*-1 proto-oncogenes. *Nature*, **359**, 554–556.

GUOHUA, P., WINPING, Z. & QIN, Z. (1981). Development of an anticomplement immunoenzyme test for the detection of EB virus nuclear antigen (EBNA) and antibody to EBNA. *J. Immunol. Meth*., **44**, 73–78.

HAMMERSCHMIDT, W. & SUGDEN, B. (1989). Genetic analysis of immortalizing functions of Epstein-Barr virus in human B lymphocytes. *Nature*, **340**, 393–397.

HENDERSON, S., ROWE, M., GREGORY, C., CROOM-CARTER, D., WANG, F., LONGNECKER, R., KIEFF, E. & RICKINSON, A.B. (1991). Induction of *bcl*-2 expression by Epstein-Barr virus latent membrane protein 1 protects infected B cells from programmed cell death. *Cell*, **65**, 1107–1115.

HENLE, W. & HENLE, G. (1979). Seroepidemiology of the virus. In *The Epstein-Barr Virus*, Epstein, M.A. & Achong, B.G. (eds), Springer-Verlag: New York, 61–78.

HETHERINGTON, C.M., CRAWFORD, D.H., BLACKLOCK, H.A., LEACH, P.A., JANOSSY, G., FRANCIS, G.E., CRAWFORD, T. & PRENTICE, H.G. (1981). Successful bone marrow transplantation with whole marrow and CFU-C depleted marrow in the marmoset (*Callithrix jacchus*). In *Bone Marrow Transplantation in Europe II*. Touraine, J.-L., Gluckman, E. & Griscelli, C. (eds), Elsevier: Amsterdam, 274–279.

HOCKENBERY, D., NUNEZ, G., MILLIMAN, C., SCHREIBER, R.D. & KORSMEYER, S.J. (1990). Bcl-2 is an inner mitochondrial membrane protein that blocks programmed cell death. *Nature*, **348**, 334–336.

HOTCHIN, N.A., READ, R., SMITH, D.G. & CRAWFORD, D.H. (1989). Active Epstein-Barr virus infection in post-viral fatigue syndrome. *J. Infect*., **18**, 143–150.

HOTCHIN, N.A., ALLDAY, M.J. & CRAWFORD, D.H. (1990). Deregulated c-*myc* expression in Epstein-Barr virus immortalized B-cells induces altered growth properties and surface phenotype but not tumorigenicity. *Int. J. Cancer*, **45**, 566–571.

KAFUKO, G.W. & BURKITT, D. (1970). Burkitt's lymphoma and malaria. *Int. J. Cancer*, **6**, 1–9.

KAWASAKI, E.S. (1990). *PCR protocols, a guide to methods and applications*. Innis, M.A., Gelfand, P.H., Sninsky, J.J. & White, T.J. (eds), Academic Press: San Diego, USA, 21–27.

KELLY, K., COCHRAN, B.H., STILES, C.D. & LEDER, P. (1983). Cell specific regulation of the c-*myc* gene by lymphocyte mitogens and platelet-derived growth factor. *Cell*, **35**, 603–610.

KIEFF, E. & LIEBOWITZ, D. (1990). Epstein-Barr virus and its replication. In *Virology: 2nd edition*. Fields, B.N. *et al*. (eds), Raven Press Ltd: New York, 1889–1920.

KRETZNER, L., BLACKWOOD, E.M. & EISENMAN, R.N. (1992). Myc and Max proteins possess distinct transcriptional activities. *Nature*, **359**, 426–428.

LAND, H., PARADA, L.F. & WEINBERG, R.A. (1983). Tumorigenic conversion of primary embryo fibroblasts requires at least two cooperating oncogenes. *Nature*, **304**, 596–602.

LAND, H., CHEN, A.C., MORGENSTEIN, J.P., PARADA, L.F. & WEINBERG, R.A. (1986). Behaviour of *myc* and *ras* oncogenes in transformation of rat embryo fibroblasts. *Molecule & Celluler Biol*., **6**, 1917–1925.

LENOIR, G.M. (1986). Role of the virus, chromosomal translocation and cellular oncogenes in the aetiology of Burkitt's lymphoma. In *The Epstein-Barr Virus: Recent Advances*. Epstein, M.A. & Achong, B.G. (eds), William Heinemann, London, 183–205.

LOMBARDI, L., NEWCOMB, E.W. & DALLA-FAVERA, R. (1987). Pathogenesis of Burkitt's lymphoma: expression of an activated c-*myc* oncogene causes the tumorigenic conversion of EBV infected human B lymphoblasts. *Cell*, **49**, 161–170.

MANIATIS, T., FRITSCH, E.F. & SAMBROOK, J. (1982). *Molecular Cloning: a Laboratory Manual*. Cold Spring Harbor Laboratory: New York, 196.

MOSS, D.J., MISKO, I.S., BURROWS, S.R., BURMAN, K., MCCARTHY, R. & SCULLEY, T.B. (1988). Cytotoxic T-cell clones discriminate between A- and B- type Epstein-Barr virus transformants. *Nature*, **331**, 719–721.

MOUGNEAU, E., LEMIEUX, L., RASSOULZADEGAN, M. & CUZIN, F. (1984). Biological activities of v-myc and rearranged c-myc oncogenes in rat fibroblast cells in culture. *Proc. Natl Acad. Sci. USA*, **81**, 5758–5762.

MILLER, G. (1979). Experimental carcinogenicity by the virus *in vivo*. In *The Epstein-Barr Virus*, Epstein, M.A. & Achong, B.G. (eds), Springer Verlag: New York, 351–372.

MURRAY, R.J., KURILLA, M.G., GRIFFIN, H.M., BROOKS, J.M., MACKETT, M., ARRAND, J.R., ROWE, M., BURROWS, S.R., MOSS, D.J., KIEFF, E. & RICKINSON, A.B. (1990). Human cytotoxic T-cell responses against Epstein-Barr virus nuclear antigens demonstrated by using recombinant vaccinia viruses. *Proc. Natl Acad. Sci. USA*, **87**, 2906–2910.

NIEDERMAN, J.C., EVANS, A.S., SUBRAHMANYAN, L. & MCCOLLUM, R.W. (1970). Prevalence, incidence and persistence of EB virus antibody in young adults. *New Engl. J. Med.*, **282**, 361–365.

PROCHOWNIK, E.V., KUKOWSKA, J. & RODGERS, C. (1988). C-myc anti-sense transcripts accelerate differentiation and inhibit G1 progression in murine erythroleukaemia cells. *Mol. Cell. Biol.*, **8**, 3683–3695.

REEDMAN, B.M. & KLEIN, G. (1973). Cellular localisation of an Epstein-Barr virus (EBV)-associated complement-fixing antigen in producer and non-producer lymphoblastoid cell lines. *Int. J. Cancer*, **11**, 499–520.

RICKINSON, A.B. (1986). Cellular immunological responses to the virus infection. In: *The Epstein-Barr Virus: Recent Advances*. Epstein, M.A. & Achong, B.G. (eds), William Heinemann, London, 75–125.

ROWE, M., ROWE, D.T., GREGORY, C.D., YOUNG, L.S., FARRELL, P.J., RUPANI, H. & RICKINSON, A.B. (1987*a*). Differences in B cell growth phenotype reflect novel patterns of Epstein-Barr virus latent gene expression in Burkitt's lymphoma cells. *EMBO J.*, **6**, 2743–2751.

ROWE, M., EVANS, H.S., YOUNG, L.S., HENNESSY, K., KIEFF, E. & RICKINSON, A.B. (1987*b*). Monoclonal antibodies to the latent membrane protein of Epstein-Barr virus reveal heterogeneity of the protein and inducible expression in virus-transformed cells. *J. Gen. Virol.*, **68**, 1575–1586.

SPENCER, C.A. & GROUDINE, M. (1991). Conrol of c-*myc* regulation in normal and neoplastic cells. *Adv. Cancer Res.*, **56**, 1–48.

SUGDEN, B., MARSH, K. & YATES, J. (1985). A vector that replicates as a plasmid and can be efficiently selected in B-lymphoblasts transformed by Epstein-Barr virus. *Mol. Cell. Biol.*, **5**, 410–413.

THOMAS, J.A., ALLDAY, M.J. & CRAWFORD, D.H. (1991). Epstein-Barr virus-associated lymphoproliferative disorders in immunocompromised individuals. *Adv. Cancer Res.*, **57**, 329–380.

THORLEY-LAWSON, D.A. & ISRAELSOHN, E.S. (1987). Generation of specific cytotoxic T-cells with a fragment of the Epstein-Barr virus encoded p63/latent membrane protein. *Proc. Natl Acad. Sci. USA*, **84**, 5384–5388.

DE THE, G., HO, H.C., KWAN, H.C., DESGRANGES, C. & FAVRE, M.C. (1970). Nasopharyngeal carcinoma (NPC) 1. Types of cultures derived from tumour biopsies and non tumorous tissues of Chinese patients with special reference to lymphoblastoid transformation. *Int. J. Cancer*, **6**, 189–206.

VAUX, D.L., CORY, S. & ADAMS, J.M. (1988). *Bcl*-2 gene promotes haemopoietic cell survival and co-operates with v-*myc* to immortalize pre-B-cels. *Nature*, **335**, 440–442.

WANG, D., LIEBOWITZ, D. & KIEFF, E. (1985). An EBV membane protein expressed in immortalized lymphocytes transforms established rodent cells. *Cell*, **43**, 831–840.

WEDDERBURN, N., EDWARDS, J.M.B., DESGRANGES, C., FONTAINE, C., COHEN, B. & DE THE, G. (1984). Infectious mononucleosis-like response in common marmosets infected with Epstein-Barr virus. *J. Infect. Dis.*, **150**, 878–882.

WESTNEAT, D.F., NOON, W.A., REEVE, H.K. & AQUADRO, C.F. (1988). Improved hybridization conditions for DNA fingerprints probed with M13. *Nucleic Acids Res.*, **16**, 4161.

WHITTLE, H.C., BROWN, J., MARSH, K., GREENWOOD, B.M., SIEPLELIN, P., TIGHE, H. & WEDDERBURN, L. (1984). T-cell control of Epsein-Barr virus-infected B cells is lost during P. *falciparum* malaria. *Nature*, **312**, 449–450.